# Surface EEG Evidence for Cerebellar Control of Distal Upper Limbs in Humans

**DOI:** 10.3390/brainsci15050440

**Published:** 2025-04-24

**Authors:** Anna Latorre, Kais Humaidan, Mauro Sanna, Maria Lucrezia Lavena, Sara Pittalis, Clio Raimondi, Elias Paolo Casula, Lorenzo Rocchi

**Affiliations:** 1 Department of Clinical and Movement Neurosciences, UCL Queen Square Institute of Neurology, University College London, London WC1N 3BG, UK; 2 Department of Medical Sciences and Public Health, University of Cagliari, 09124 Cagliari, Italy; humaidankais@gmail.com (K.H.); m.sanna@protonmail.com (M.S.); malulavena@gmail.com (M.L.L.); sarapittalis01@gmail.com (S.P.); clioraimondi@gmail.com (C.R.); l.rocchi@ucl.ac.uk (L.R.); 3 Department of System Medicine, “Tor Vergata” University of Rome, Via Montpellier 1, 00133 Rome, Italy; elias.casula@gmail.com

**Keywords:** cerebellum, electroencephalography, electrocerebellogram, motor cortex, alpha rhythm, tremor, sinusoidal movements

## Abstract

**Background/Objectives**: The cerebellum plays a crucial role in motor control, but its direct electrophysiological investigation in humans is challenging. Electrocerebellograms (ECeGs), recorded via surface electrodes below the inion, have been proposed as a non-invasive method to assess cerebellar activity. However, its interpretation is complicated by potential interference from occipital alpha rhythms and neck muscle signals. This study aimed to investigate whether ECeG signals genuinely reflect cerebellar involvement during upper limb movement and to explore possible confounding influences. **Methods**: We recorded electroencephalograms (EEGs) from occipital (Oz) and cerebellar electrodes (Cb1 and Cb2), alongside EMGs from forearm muscles in healthy individuals performing sinusoidal (~1 Hz) and tremor-like (~4 Hz) wrist movements. To assess occipital contamination, recordings were obtained under both eyes-open and eyes-closed conditions. **Results**: Occipital alpha power was present in Cb1 and Cb2 but was less affected by eye-opening than at Oz, suggesting a partially distinct neural source. During the tremor condition, movement-frequency power increased at Cb2 and C3 (corresponding to the ipsilateral cerebellar hemisphere and contralateral motor cortex), indicating authentic cerebellar activity. No significant movement-related EEG changes were observed during sinusoidal movements, likely due to weaker neuronal synchronization. **Conclusions**: These findings suggest that ECeGs can detect cerebellar signals linked to movement, especially during faster and rhythmic motions, and are only moderately affected by occipital contamination. This supports the feasibility of non-invasive cerebellar electrophysiology and underscores the need for further methodological refinement to enhance signal specificity.

## 1. Introduction

The cerebellum is a crucial structure for human motor control, playing a fundamental role in coordinating voluntary movements, maintaining balance, and fine-tuning motor output. It achieves this by integrating sensory information with motor commands, ensuring the smooth and precise execution of movements [1,2]. Beyond its classical role in motor coordination, the cerebellum also contributes to motor learning [3], error correction, and even certain cognitive processes [4]. Despite extensive knowledge gained through neuroanatomical and functional imaging studies, the direct electrophysiological investigation of cerebellar activity in humans remains challenging. This is due to a number of physical and physiological factors, including a larger distance between the cerebellar cortex and the scalp, the “closed field” geometry of Purkinje cells, low synchrony in neuronal firing, and high-frequency activity [5,6,7,8,9]. Consequently, the cerebellum in intact humans has been mostly investigated with indirect methods, such as classical eyeblink conditioning [10], cerebellar-brain inhibition [11,12,13], and cerebellar evoked potentials [14,15], the latter two assessed by means of transcranial magnetic stimulation.

Recent studies have suggested the possibility of the direct, non-invasive recording of cerebellar activity using scalp electrodes placed below the inion. The signal obtained in this way, called an electrocerebellogram (ECeG) [16,17], is thought to reflect cerebellar local field potentials. Most of the previous literature suggests that the ECeG is mostly generated by the vestibular portion of the cerebellum. Evidence includes the modulation of an ECeG by optokinetic, vection, and vestibular stimulation; mastoid taps; and auditory tones [16,18,19,20,21,22,23,24]. ECeG oscillations in the theta frequency band have also been demonstrated to be relevant to the pathophysiology of postural instability and the freezing of gait in Parkinson’s disease [25,26], suggesting the possible role of this activity in postural control.

The possibility of non-invasively recording cerebellar electrical activity linked to the control of upper limb movements is more controversial. Indirect evidence comes from the observation of increased ECeG power in patients with essential tremor [27,28]. Studies on healthy subjects suggest the existence of movement-related potentials of cerebellar origin during ballistic movements of the finger [29,30]. However, two key limitations hinder the interpretation of these studies. First, the causal relationship between ECeG signals and the electromyographic (EMG) activity of upper limb muscles remains unclear, as previous studies have not systematically examined the temporal and functional correlation between these signals. Second, the close anatomical proximity of the cerebellum to the occipital cortex raises concerns regarding the contamination of ECeG recordings by occipital alpha activity (8–12 Hz), a dominant rhythm that could confound the interpretation of cerebellar oscillatory dynamics.

The present study aims to address these limitations by systematically assessing an ECeG during rhythmic upper limb movements under different eye closure conditions. By simultaneously recording ECeGs and EMGs, we seek to clarify the relationship between cerebellar oscillatory activity and muscle activation. Furthermore, by manipulating eye closure conditions, we aim to disentangle genuine cerebellar contributions from potential contamination caused by occipital alpha activity. Overall, our findings indicate that it is possible to record ECeG oscillations at the same frequency as upper limb EMG activation and that, in resting conditions, an ECeG is only partially contaminated by the occipital alpha rhythm. These results were obtained with a limited number of electrodes, indicating the possibility of studying cerebellar electrical activity involved in upper limb motor control even with limited resources.

## 2. Materials and Methods

### 2.1. Participants

Sixteen right-handed healthy subjects [31] (age 28.2 ± 5.7, eight females) participated in this study. The subjects had no history of neurological or psychiatric disorders and were not taking drugs active in the central nervous system. All the procedures were performed in accordance with the Declaration of Helsinki and were approved by the local review board (ID PG/2018/8829). Participants gave written informed consent prior to the experimental session.

### 2.2. Experimental Design

**Figure 1:**
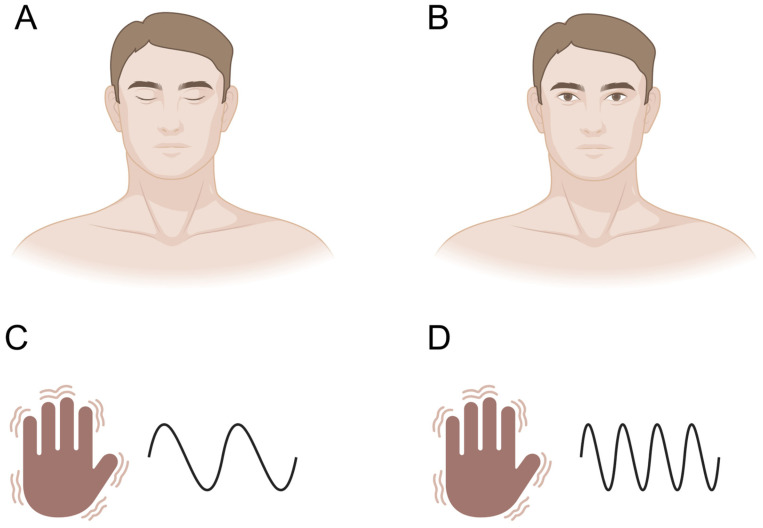
Experimental blocks. (**A**) Resting EEG with eyes closed. (**B**) Resting EEG with eyes open. (**C**) Sinusoidal movements of the wrist. (**D**) Simulated tremor. See text for details.

Subjects were placed supine on a couch with a pillow under the head to maximize comfort and reduce tonic contraction of the neck muscles. Four different recording blocks of 90 s duration were performed in a single experimental session (Figure 1).

Block 1 (“Rest-eo”) was performed during rest with eyes open; block 2 (“Rest-ec”) was similar to the previous block, but subjects kept their eyes closed. These first two blocks were conducted to assess the possible contamination of cerebellar activity generated by the occipital cortex due to volume conduction. Activity in the alpha frequency band was used as an indicator, as this is mainly generated in visual cortices and it is partially suppressed by eye-opening, and reaches its maximum expression with eyes closed. Blocks 3 and 4 were recorded with eyes open. In block 3 (“Sinus”), subjects had to perform flexion–extension sinusoidal movements with their right wrist at a frequency of approximately 1 Hz; lastly, block 4 (“Tremor”) was similar to block 3, except that the flexion–extension wrist movements were performed at the highest possible frequency, with the aim of simulating a tremor. The aim of the last two blocks was to assess the possible effect of voluntary, rhythmic movement on cerebellar activity.

### 2.3. Data Recording, Analysis, and Statistics

**Figure 2:**
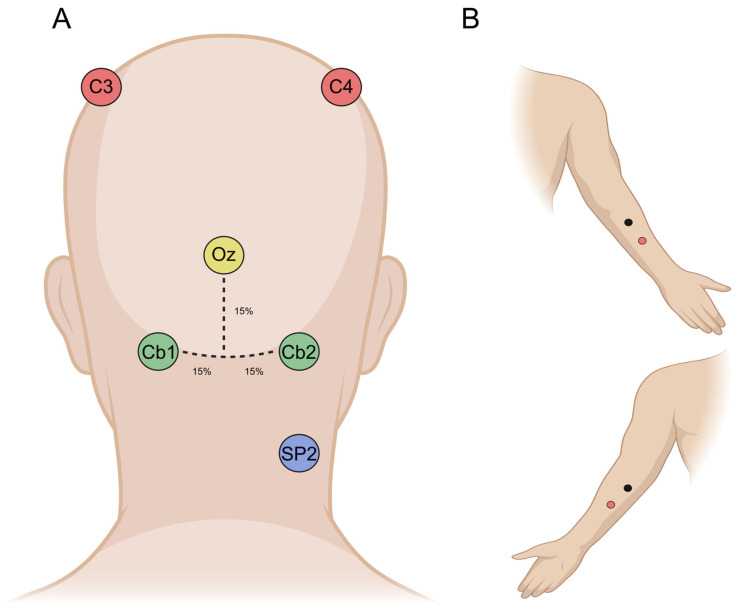
Placement of recording electrodes on the scalp (**A**) and the forearm (**B**). See text for details.

After careful skin preparation and the application of conductive gel, the following six Ag/AgCl electrodes were placed on the participants’ scalps (Figure 2): three cerebral electrodes (C3, C4, and Oz) positioned according to the International 10–20 System [32], two cerebellar electrodes (Cb1 and Cb2), and one electrode over the right splenius muscle (SP2) to assess potential muscle contributions to cerebellar signals. Besides ECeG studies conducted on animal models [33], there have been studies on humans that have provided data with good spatial resolution and localization, offering valuable insights into ECeGs and electrode positions directly on the human brain [6].

Accordingly, the reference electrode was positioned at Fpz. Based on the previous literature [16], Cb1 and Cb2 were positioned approximately 15% below Oz and 15% laterally from the midline, on the left and right side, respectively. This location corresponds to the lower part of the posterior cerebellum, approximately in the medial portion of lobules VII, VIII, and IX, where the bone thickness is expected to be smaller compared to the inion [16]. Additionally, during the motor tasks, four muscle electrodes (M1 and M2, with their respective references) were placed over the flexor and extensor carpi radialis, respectively. The active electrodes were positioned at the center of the muscle belly, while the reference electrodes were placed approximately 3 cm distally. Cerebral and cerebellar signals were amplified with a gain of 50,000, using a high-pass filter at 0.5 Hz and a low-pass filter at 1000 Hz. Muscle signals were amplified with a gain of 1000, applying a high-pass filter at 1 Hz and a low-pass filter at 1000 Hz. Recording was performed with a Digitimer D360 (Digitimer Ltd., Welwyn Garden City, UK), while a CED 1401 A/D laboratory interface (Cambridge Electronic Design Ltd., Cambridge, UK) was used for digitization (1000 Hz sampling frequency). Data were visualized online with the Signal software Version 7.06 (Signal software, Cambridge Electronic Design, Cambridge, UK) and stored on a computer for further analysis.

Signal analysis was performed with MATLAB (Version 2020a, MathWorks Inc., Natick, MA, USA). Statistical analyses were carried out with version 26.0 (IBM Corp, Armonk, NY, USA). To exclude biases due to the effect of neck muscle contraction, a one-way analysis of variance (ANOVA) with the factor “Condition” (Rest-eo, Rest-ec, Sinus, and Tremor) was performed on the root mean square (RMS) of the EMG recorded from electrode SP2. For all electroencephalographic and EMG signals, the power spectral density (PSD) was calculated using Welch’s periodogram [34]. We used a Hann window to double the length of the sampling rate (2s) to ensure a good tradeoff between averaging power across different segments and frequency resolution. We set the latter at 0.1 Hz to accurately resolve the EEG dynamics of interest, which were around 10 Hz frequency.

**Figure 3:**
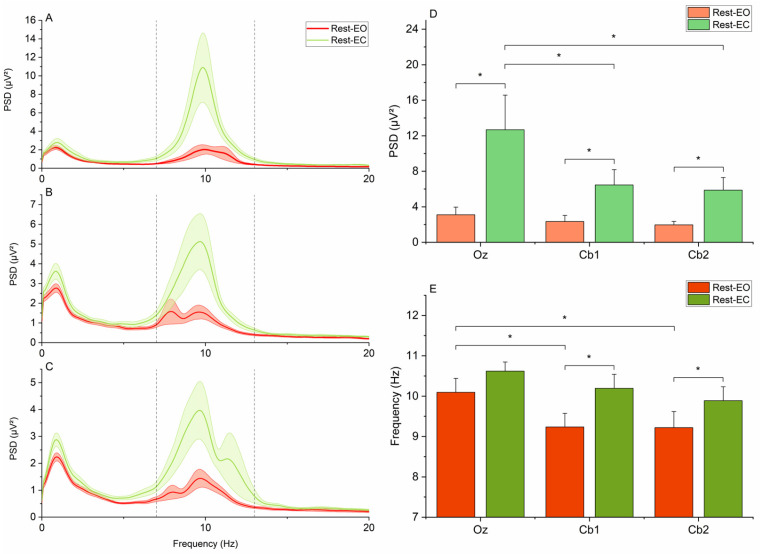
Alpha power and frequency. (**A**–**C**) depict the PSD at electrodes Oz, Cb1, and Cb2, respectively, in Rest-eo (red lines) and Rest-ec (green lines) conditions. Shaded areas indicate the standard error of the mean. The black vertical dashed lines indicate the frequency range from which values of alpha power and frequency were extracted (see text for details). (**D**,**E**) indicate pairwise comparisons for values of alpha power and frequency, respectively. Average values (bars) and standard errors of the mean (whiffs) are depicted. Brackets and asterisks indicate statistically significant differences (*p* < 0.05).

One of the main aims of the present study was to assess a possible overlap of EEG signals recorded from Oz and Cb1-Cb2. To this end, we extracted power in the alpha frequency band in each subject by selecting the maximum values of the PSD in a frequency window ranging from 7 to 13 Hz (Figure 3A–C) from electrodes Oz, Cb1, and Cb2 during recording blocks performed at rest with eyes open and closed (Rest-eo and Rest-ec conditions). To verify the possible contamination of cerebellar activity by the occipital alpha rhythm, a two-way repeated measure (RM) ANOVA with the factors “Electrode” (Oz, Cb1, and Cb2) and “Condition” (Rest-eo and Rest-ec) was performed on the alpha power values. We hypothesized that if the recorded cerebellar activity was completely due to contamination from occipital sources via volume conduction, we would observe no differences in alpha power across electrodes; by contrast, significant differences in alpha power would point to the possibility that Cb1 and Cb2 are capturing cerebellar activity. An ANOVA with the same factors and similar assumptions was also performed on the frequency of the alpha peak.

Peak EMG frequency was selected in each subject and recording condition (Tremor and Sinus) as the maximum PSD value. Values were entered in a two-way RM-ANOVA with “Muscle” (M1 and M2) and “Task” (Sinus and Tremor) as the factors of analysis. The aim was twofold, as we sought to confirm (1) that movement frequency was higher in the “Tremor” compared to the “Sinus” condition and that (2) the frequency of activation in the two muscles was the same. The latter is important since, in the following analyses, the average of the two was used to extract power from the EEG.

Lastly, we tested whether movement induced an increase in EEG power corresponding to the frequency of EMG activation. To do so, we performed two two-way RM ANOVAs. In the first, the factors “Condition” (Rest-eo and Sinus) and “electrode” (C3, C4, Oz, Cb1, and Cb2) were used, while in the second, the level “Sinus” within the first factor was replaced by “Tremor”. The normality of distribution was assessed with the Shapiro–Wilk test, whereas Greenhouse–Geisser correction was used, if necessary, to correct for non-sphericity (i.e., Mauchly’s test < 0.05). *p*-values < 0.05 were deemed significant. Bonferroni’s post hoc test was used for post hoc comparisons following ANOVAs.

## 3. Results

The Shapiro–Wilk test yielded non-significant results for all distributions. There were no significant differences in RMS values obtained by signals recorded at the SP2 electrode. This was confirmed by the one-way ANOVA, which showed the non-significant main effect of “Condition” (F_3,45_ = 0.704, *p* = 0.555).

The two-way RM-ANOVA of alpha power showed the significant main effects of “Condition” (F_1,15_ = 8.391, *p* = 0.011) and “Electrode” (F_2,30_ = 5.083, *p* = 0.013), as well as a significant “Condition × Electrode” interaction (F_2,30_ = 3.836, *p* = 0.033). Post hoc comparisons showed that, in all electrodes, alpha power was higher in the Rest-ec condition than the Rest-eo condition (Oz: 12.68 ± 3.89 vs. 3.11 ± 0.85 µV^2^, *p* = 0.019; Cb1: 6.45 ± 1.73 vs. 2.34 ± 0.68 µV^2^, *p* = 0.022; Cb2: 5.87 ± 1.42 vs. 1.97 ± 0.38 µV^2^, *p* = 0.011). There were also no statistically significant differences in alpha power between electrodes in the Rest-eo condition (Oz vs. Cb1: 3.11 ± 0.85 vs. 2.34 ± 0.68 µV^2^, *p* = 0.338; Oz vs. Cb2: 3.11 ± 0.85 vs. 1.97 ± 0.38 µV^2^, *p* = 0.154; Cb1 vs. Cb2: 2.34 ± 0.68 vs. 1.97 ± 0.38 µV^2^, *p* = 0.430). However, in the Rest-ec condition, alpha power became significantly higher in Oz compared to the other two electrodes (Oz vs. Cb1: 12.68 ± 3.89 vs. 6.45 ± 1.73 µV^2^, *p* = 0.03; Oz vs. Cb2: 12.68 ± 3.89 vs. 5.87 ± 1.42 µV^2^, *p* = 0.044; Cb1 vs. Cb2: 6.45 ± 1.73 vs. 5.87 ± 1.42 µV^2^, *p* = 0.430), while the difference between Cb1 and Cb2 remained non-significant (Cb1 vs. Cb2: 6.45 ± 1.72 vs. 5.87 ± 1.42 µV^2^, *p* = 0.430) (Figure 3).

The two-way RM-ANOVA of alpha frequency showed the significant main effects of “Condition” (F_1,15_ = 6.283, *p* = 0.024) and “Electrode” (F_2,30_ = 8.556, *p* = 0.001), as well as a non-significant “Condition × Electrode” interaction (F_2,30_ = 0.305, *p* = 0.739). Post hoc comparisons showed that, in the Rest-eo condition, the peak alpha frequency was higher in electrode Oz compared both to electrodes Cb1 (10.09 ± 0.35 vs. 9.23 ± 0.34 Hz, *p* = 0.021) and Cb2 (10.09 ± 0.35 vs. 9.22 ± 0.39 Hz, *p* = 0.032), whereas no significant differences between the electrodes were found in the Rest-ec condition (all *p*-values > 0.05). Notably, while eye closure had no significant effect on alpha frequency recorded from Oz (10.09 ± 0.35 vs. 10.61 ± 0.23 Hz, *p* = 0.282), it significantly increased it both at Cb1 (9.23 ± 0.34 vs. 10.19 ± 0.35 Hz, *p* = 0.032) and Cb2 (9.21 ± 0.39 vs. 9.88 ± 0.34 Hz, *p* = 0.041) (Figure 3).

**Figure 4:**
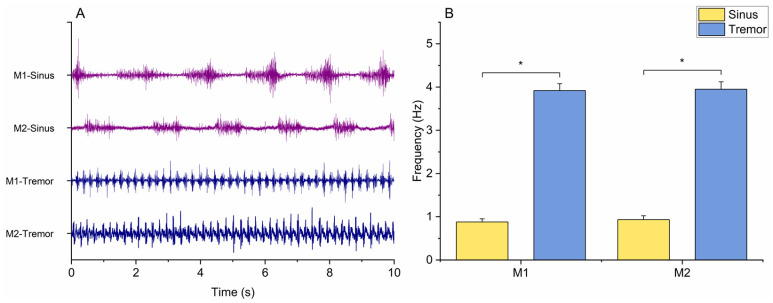
In (**A**), EMG traces from both muscles and recording conditions (Sinus and Tremor) in a representative subject are depicted. (**B**) shows EMG frequency in Sinus and Tremor conditions. Average values (bars) and standard errors of the mean (whiffs) are depicted. M1: flexor carpi radialis; M2: extensor carpi radialis. Brackets and asterisks indicate statistically significant differences (*p* < 0.05).

The two-way RM ANOVA of EMG frequency showed the non-significant main effect of “Muscle” (F_1,15_ = 0.878, *p* = 0.364), the significant main effect of “Task” (F_1,15_ = 306.296, *p* < 0.001), and a significant “Muscle × Task” interaction (F_1,15_ = 0.042, *p* = 0.841). Post hoc comparisons indicated a significantly higher activation frequency in the “Tremor” condition compared to “Sinus”, both for M1 (3.91 ± 0.16 vs. 0.88 ± 0.07 Hz, *p* < 0.001) and M2 (3.95 ± 0.17 vs. 0.93 ± 0.09 Hz, *p* < 0.001). By contrast, peak EMG frequency was not significantly different between M1 and M2 in both tasks (all *p*-values > 0.05) (Figure 4).

**Figure 5:**
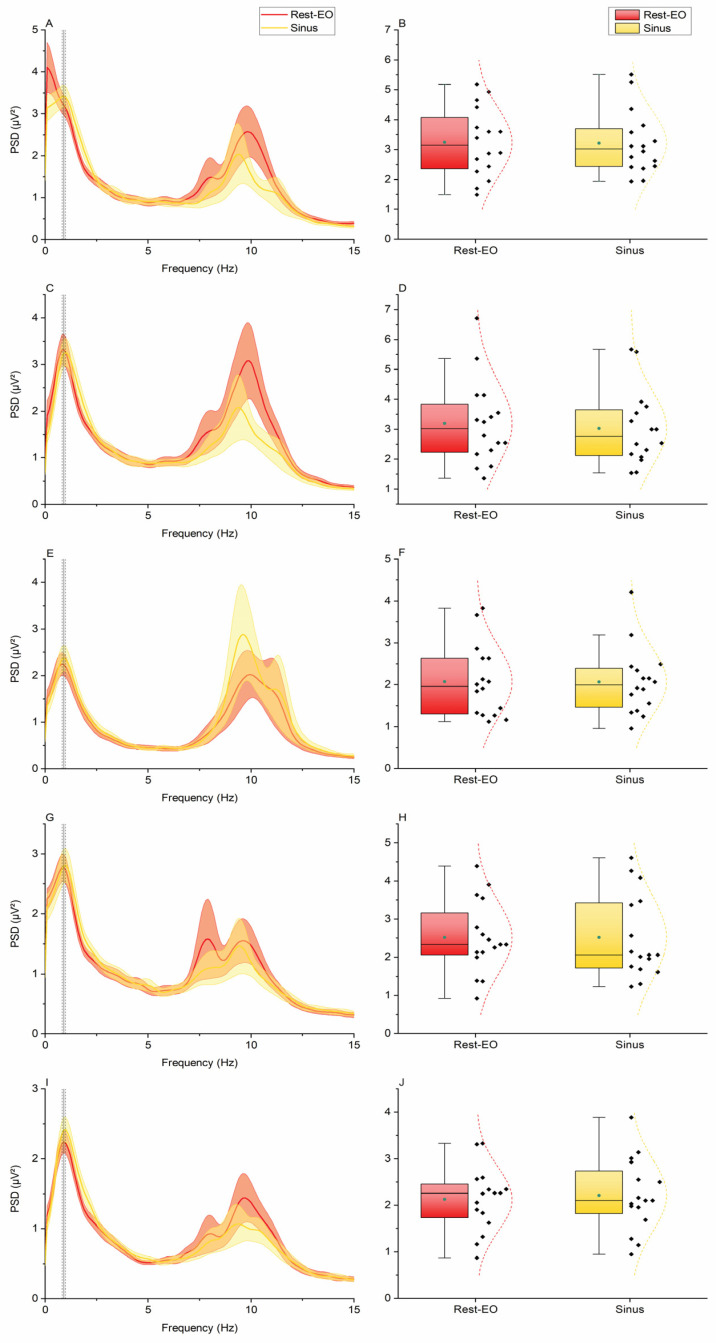
ECeG results from the “Sinus” condition. (**A**,**C**,**E**,**G**,**I**) PSD from each recording electrode (C3, C4, Oz, Cb1, and Cb2, respectively). Vertical black lines indicate the average EMG activity of the related condition (“Sinus”) while vertical dashed black lines indicate the corresponding standard error. Shaded areas indicate the standard error of the ECeG PSD. (**B**,**D**,**F**,**H**,**J**) Box plots of power values extracted from the PSDs corresponding to peak EMG activity. Box edges represent the standard deviation, vertical bars are the 25° and 75° percentiles, horizontal black lines are the medians, green dots are the averages, and dashed curves represent the gaussian fitting of the data.

**Figure 6:**
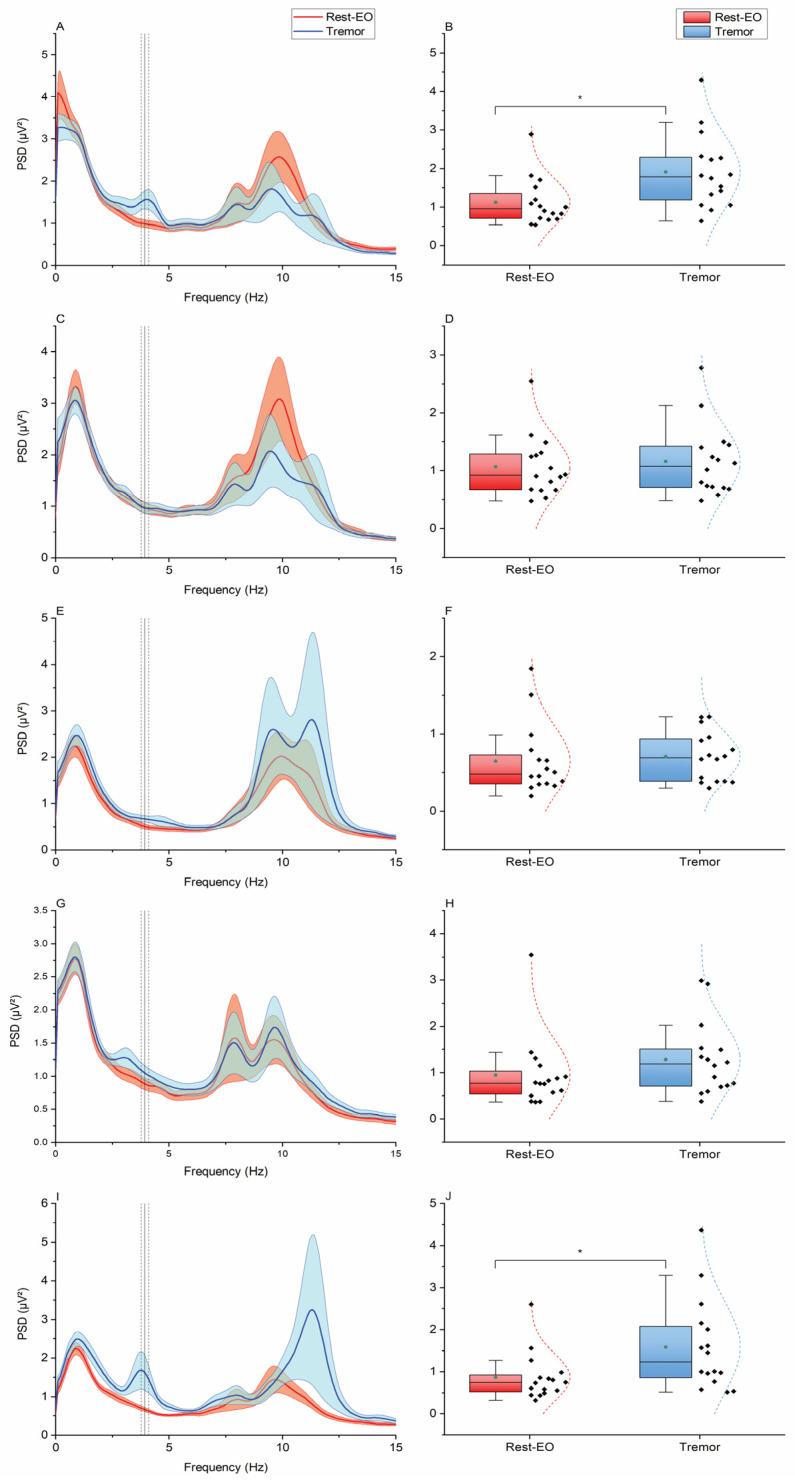
ECeG results from the “Tremor” condition. (**A**,**C**,**E**,**G**,**I**): PSD from each recording electrode (C3, C4, Oz, Cb1, and Cb2, respectively). Vertical black lines indicate the average EMG activity of the related condition (“Tremor”), while vertical dashed black lines indicate the corresponding standard error. Shaded areas indicate the standard error of the ECeG PSD. (**B**,**D**,**F**,**H**,**J**): Box plots of power values extracted from the PSDs corresponding to peak EMG activity. Box edges represent the standard deviation, vertical bars are the 25° and 75° percentiles, horizontal black lines are the medians, green dots are the averages, and dashed curves represent the gaussian fitting of the data. Brackets and asterisks indicate statistically significant differences (*p* < 0.05).

The two-way ANOVA of EEG power at the EMG activation frequency in the “Sinus” task showed the non-significant main effect of “Condition” (F_1,15_ = 0.024, *p* = 0.879), the significant main effect of “Electrode” (F_4,60_ = 14.207, *p* < 0.001), and a non-significant “Condition × Electrode” interaction (F_4,60_ = 0.494, *p* = 0.740). Post hoc comparisons showed that EEG power at the “Sinus” EMG frequency was smaller at electrode Oz compared to C3 and C4, and this was true both for the “Rest-eo” (Oz vs. C3: 2.07 ± 0.21 µV^2^ vs. 3.24 ± 0.29 µV^2^, *p* < 0.001; Oz vs. C4: 2.07 ± 0.21 µV^2^ vs. 3.19 ± 0.35 µV^2^, *p* < 0.001) and “Sinus” conditions (Oz vs. C3: 2.07 ± 0.20 µV^2^ vs. 3.22 ± 0.26 µV^2^, *p* < 0.001; Oz vs. C4: 2.07 ± 0.20 Hz vs. 3.02 ± 0.31 Hz, *p* < 0.001). However, there were no significant changes at each electrode level when comparing the “Rest-eo” and “Sinus” conditions (all *p*-values > 0.05) (Figure 5). By contrast, the two-way ANOVA of EEG power at the EMG activation frequency in the “Tremor” task showed the significant main effect of “Condition” (F_1,15_ = 9.778, *p* = 0.007), the significant main effect of “Electrode” (F4,60 = 5.876, *p* < 0.001), and a significant “Condition × Electrode” interaction (F_4,60_ = 4.051, *p* = 0.006). Post hoc comparisons showed that compared to “Rest-eo”, EEG power was higher in the “Tremor” condition in electrodes C3 (1.12 ± 0.15 vs. 1.91 ± 0.24 µV^2^, *p* = 0.017) and Cb2 (0.78 ± 0.13 vs. 1.90 ± 0.49 µV^2^, *p* = 0.035) (Figure 6).

## 4. Discussion

The findings of the present study suggest that electrodes placed below the inion in healthy individuals can indeed detect cerebellar electrical activity. While the occipital alpha rhythm remains a potential confound, the differential effect of eye closure on signals recorded from cerebellar electrodes compared to Oz supports the presence of genuine cerebellar contributions to at least part of the recorded activity. This conclusion is further reinforced by the observed increase in ECeG power at movement frequency, suggesting a link between cerebellar oscillations and motor activity.

### 4.1. Contamination of ECeGs by Neck Muscle Activity and Occipital Alpha Rhythm

A key objective of this study was to assess two potential confounding factors that could hinder the reliable recording of ECeGs. The first was neck muscle activation, given that Cb1 and Cb2 electrodes are positioned near the splenius capitis and semispinalis capitis, muscles whose activity can overlap with ECeG signals, particularly in the higher-frequency range. To address this, EMGs were recorded using an SP2 electrode placed below Cb2 (Figure 2). EMG activity was continuously monitored across all experimental blocks to determine whether differences in muscle activation might influence cerebellar recordings. The absence of significant variations in EMG activity across conditions suggests that muscle activity did not substantially influence our results. Although only one electrode was placed on the neck due to technical constraints, we believe it provides a representative measure of overall neck extensor activity, as these muscles function synergistically [35]. Moreover, the most prominent findings were observed in ECeG signals recorded from Cb2, supporting the decision to use SP2 alone rather than a combination of SP1 and SP2 (see below).

Another potential confound in ECeG recordings is contamination by the occipital alpha rhythm. Given the anatomical proximity between the occipital cortex and the cerebellum, volume conduction could lead to partial overlap between occipital and cerebellar signals [36]. Indeed, the presence of occipital influences in Cb1 and Cb2 recordings is suggested by the reduction in alpha power observed across all three electrodes (Oz, Cb1, and Cb2) in the Rest-eo condition compared to Rest-ec (Figure 3A–C), consistent with the well-documented suppression of occipital alpha activity following eye-opening [37]. However, a key finding is that this effect is not uniform across electrodes: the reduction in alpha power is significantly greater at Oz than at the cerebellar electrodes. Bivariate comparisons further confirmed a significant difference in alpha power between Oz and both Cb1 and Cb2 in the Rest-ec condition (Figure 3D). If the signals recorded at Cb1 and Cb2 were purely of occipital origin, eye closure would be expected to have a comparable effect on all three electrodes. Instead, the observed differences suggest that while some degree of occipital contamination is present, it does not fully account for the signals recorded at the cerebellar electrodes. This leaves open the possibility that at least part of the ECeG signal originates from the cerebellum itself.

A similar analysis was conducted on the peak frequency of the alpha rhythm, yielding results that are somewhat complex to interpret. While no significant differences were observed in peak alpha frequency across Oz, Cb1, and Cb2 under the Rest-ec condition, in the Rest-eo block, Oz exhibited a significantly higher peak frequency than the cerebellar electrodes (Figure 3E). This finding supports the notion that signals recorded at Cb1 and Cb2 have at least partially distinct sources compared to Oz. However, the underlying mechanism remains unclear. One possible explanation is that the cerebellar electrodes capture a mixture of occipital alpha and lower-frequency cerebellar activity, particularly in the theta band. The summation of these components could result in an overall reduction in the observed peak frequency at Cb1 and Cb2. This hypothesis is consistent with prior studies reporting prominent cerebellar theta-band activity in both animal models [38] and humans [39]. Further supporting this interpretation, we found that while eye-opening had no significant effect on the peak alpha frequency recorded at Oz, it led to a significant decrease at Cb1 and Cb2. This suggests that, as occipital alpha power diminishes with eye-opening, the influence of underlying cerebellar theta activity becomes more apparent.

### 4.2. Relationship Between the ECeGs and Rhythmic Upper Limb Voluntary Movements

The analysis of EMG activity from the forearm flexor and extensor muscles revealed a significantly lower frequency in the Sinus condition compared to the Tremor condition (Figure 4B). Notably, no significant differences were found between the agonist and antagonist muscles within each task. This finding is particularly relevant, as the average frequency of both muscles was subsequently used for EEG power extraction.

In the Sinus condition, no significant differences in power at the movement frequency were observed (Figure 5). In contrast, the Tremor condition yielded noteworthy results, with significant power increases detected in both cortical and cerebellar electrodes. Specifically, power increases corresponding to the movement frequency were observed at C3 and Cb2—electrodes positioned over the motor cortex contralateral to the moving limb and the cerebellar hemisphere ipsilateral to it (i.e., the right upper limb) (Figure 6). This finding aligns with established neuroanatomy, where the motor cortex governs voluntary movements of the contralateral limbs and receives projections from the contralateral cerebellar hemisphere via the cerebellar-thalamo-cortical tract [40,41].

The disparity in results between the two movement conditions may be explained by both methodological and physiological factors. From a methodological standpoint, the known 1/f distribution of EEG PSDs results in a decline in power at higher frequencies [42,43]. Consequently, the naturally higher power at lower frequencies may obscure activity peaks at low frequencies, such as the ~1 Hz frequency of the sinusoidal movements in this study (Figure 5). From a physiological perspective, the characteristics of sinusoidal movement—slow and relatively weak—may fail to generate sufficient neuronal synchronization to be detectable at the scalp level. In contrast, the simulated tremor at ~4 Hz, which involves more forceful and rapid muscle contractions, likely enhances neuronal synchronization at the movement frequency, making it more detectable in surface EEG recordings. Indeed, it is well established that cortical neuronal firing rates scale with movement velocity and force. Similarly, neuroimaging studies have demonstrated increased cerebellar activation with higher movement velocities and frequencies [1,44,45]. Furthermore, tremor-like movements necessitate rapid alternating contractions of agonist and antagonist muscles to regulate movement amplitude—a process in which the cerebellum plays a crucial role. Animal studies [46,47,48] and research in healthy humans and patients with cerebellar dysfunction [49,50] have highlighted the cerebellum’s essential contribution to the precise timing of antagonist and agonist muscle activation during rapid movements. While further research is needed to fully elucidate these mechanisms, our findings from the tremor condition support the feasibility of detecting cerebellar activity synchronized to movement frequency using surface electrodes.

## 5. Limitations and Conclusions

In conclusion, our study provides evidence that surface EEG electrodes positioned below the inion can detect cerebellar activity related to upper limb movement. While occipital alpha rhythms contribute to ECeG recordings, differences in eye closure effects and movement-related power increases suggest a genuine cerebellar component. Notably, tremor-like movements at ~4 Hz elicited clear cerebellar and cortical synchronization, whereas slower sinusoidal movements did not.

Despite these promising findings, one important limitation of our study is the relatively small sample size, which may affect the generalizability of the results and the statistical power of the study, potentially making it more difficult to detect subtle but meaningful effects. This could contribute to non-significant findings that might reflect limited power rather than a true absence of effect. Larger, more diverse cohorts are needed to validate and extend these findings across different populations and experimental conditions. Additionally, the use of a limited number of electrodes to capture cerebellar activity was constrained by the limited number of channels available in our amplifier. However, we note that the number of electrodes employed is comparable to that used in most studies in the field. Finally, although only one electrode was placed on the neck, we believe it provides a representative measure of overall neck extensor activity, as these muscles function synergistically. Notably, the most prominent findings were observed in ECeG signals recorded from Cb2, supporting the choice of using SP2 alone as a reasonable and effective approach [35].

Our results carry significant implications for future applications, especially in the context of action tremor disorders such as essential tremor. These conditions are believed to arise from abnormal oscillatory activity within the cerebellum, yet direct electrophysiological evidence in humans remains scarce. The ability to non-invasively monitor cerebellar rhythms using ECeGs offers a promising avenue for better understanding the pathophysiology of these disorders. Furthermore, it may eventually inform the development of diagnostic tools, treatment monitoring, and even neuromodulation strategies aimed at targeting dysfunctional cerebellar circuits.

These findings support the feasibility of non-invasive cerebellar electrophysiology and highlight the need for further studies to refine recording techniques and clarify the functional significance of ECeG in motor control.

## Data Availability

The data presented in this study are available upon request from the corresponding author. The data are not publicly available due to privacy restrictions.

## Abbreviations

ECeG	Electrocerebellogram
EEG	Electroencephalogram
EMG	Electromyography
PSD	Power spectral density
RMS	Root mean square
